# Miniature Coil for Wireless Power and Data Transfer through Aluminum †

**DOI:** 10.3390/s21227573

**Published:** 2021-11-15

**Authors:** Juan M. Romero-Arguello, Anh-Vu Pham, Christopher S. Gardner, Brad T. Funsten

**Affiliations:** 1 Department of Electrical and Computer Engineering, University of California Davis, Davis, CA 95616, USA; ahpham@ucdavis.edu; 2 Lawrence Livermore National Laboratory, Livermore, CA 95616, USA; gardner36@llnl.gov (C.S.G.); funsten1@llnl.gov (B.T.F.)

**Keywords:** inductive coupling, wireless power, inductive power transfer

## Abstract

This paper presents the design and development of miniature coils for wireless power and data transfer through metal. Our coil has a total size of 15 mm × 13 mm × 6 mm. Experimental results demonstrate that we can harvest 440 mW through a 1 mm-thick aluminum plate. Aluminum and stainless-steel barriers of different thicknesses were used to characterize coil performance. Using a pair of the designed coils, we have developed a through-metal communication system to successfully transfer data through a 1 mm-thick aluminum plate. A maximum data rate of 100 bps was achieved using only harvested power. To the best of our knowledge, this is the first report that demonstrates power and data transfer through aluminum using miniature coils.

## 1. Introduction

Wireless power and data transfer through metal enables a new set of applications in critical fields such as sensing of temperature and pressure behind sealed metal containers in aircraft fuselages, vehicle armor, and isolated chambers [1,2,3,4]. Inductive coupling is key to non-contact or wireless power and data transfer through metal surfaces [5,6]. Research in the area of wireless power and data transfer through metal has been mainly focused on large coils for power transfer [7,8]. In [9], authors employed a coil with an outer diameter of 30 mm enclosing a metal pipe to transfer 30 µW through stainless steel (1.6-mm). In [4], a solenoid coil of 234 mm × 63 mm × 61 mm was used with an even larger core to transfer 1.2 W at 10% efficiency through stainless steel (10-mm). In [10,11,12], a helical stacked coil of 49 mm outer diameter and 50 mm height demonstrated power transfer of 5 W at 9% efficiency through aluminum (3.1 mm). Only [9] has partially demonstrated power and data transfer through metal using the same coil structure, but information on the coil size and data rate were not available. In general, the size of a coil used for wireless power transfer through metal ranges between 30 mm to 400 mm [4,9,10,11,12,13]. The large coil size poses a limitation for certain applications [14].

This journal article is an extended version of the paper “Miniature Coil Design for Through Metal Wireless Power Transfer” presented in the 2021 IEEE Wireless Power Transfer Conference, San Diego, CA, USA, 1–4 June 2021 [15]. In our conference paper, we demonstrated the optimization of a wire gauge for building a planar circular geometry coil. The planar circular geometry coils were used in an inductive link to demonstrate 100 mW harvested power through a 1 mm-thick aluminum plate without any data communication.

In this paper, we present the development of a miniature coil for through metal power and data transfer using inductive coupling. Our new coil has a very small size of 15 mm × 13 mm × 6 mm. Our developed coil deviates from the traditional flat circular or square geometries [16] to maximize the effect of its ferrite core to obtain superior harvested power in through metal scenarios. To fabricate the coil, 24 AWG copper wire was tightly wound around a rectangular ferrite core. The winding covered the entire core, with more than 25 turns per layer and four layers in total. Two identical coils placed on both side of a 1 mm-thick aluminum plate were used to form an inductive link for energy transfer. The experimental results demonstrate that 440 mW was harvested through the 1 mm aluminum plate using our miniature coils. The coil can operate up to 2 kHz for aluminum and stainless steel metal barriers. We report the measured harvested power and efficiency of the new coil for stainless steel and aluminum barriers with different thicknesses. We developed a through-metal communication system where the same pair of coils is used for both energy transfer and wireless communications. Furthermore, the inside communication subsystem (inside a metal box) only uses harvested power for operation. We have demonstrated a successful bidirectional communication at a minimum of 100 bps using 75 mW harvested energy for the inside subsystem through a 1 mm aluminum barrier. Communication through aluminum is significant since is a light-weight material.

## 2. Miniature Coil

The miniature coil design is based on the principle of maximizing the interaction between the coil winding and its core [17]. To maximize this interaction, the coil is designed by winding a copper wire around a ferrite core, instead of the traditional planar coil geometries in our previous work [15]. Using this type of geometry, the flux path is reduced to about 50% of the coil length to minimize the overall size of the required coil [18]. As a result, we are able to design a very compact coil with very high inductance and quality factor even at a very low operating frequency.

**Figure 1:**
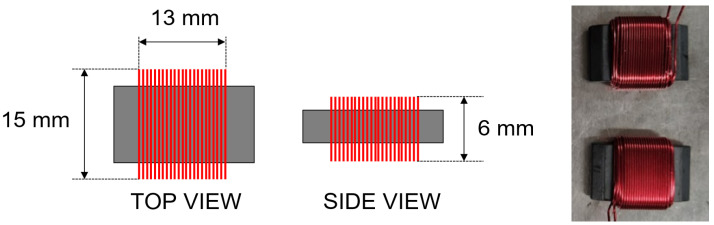
Diagram of designed and fabricated coil.

Figure 1 shows the diagram and photograph of the fabricated coils. The coil is made using insulated 24 AWG copper wire, 25 turns per layer, and 4 layers in total. The core usable area is 13 mm × 13 mm and made of Material 43 from Fair-Rite with $\mu_{i}=800$ [19]. The use of ferrite will help increase the self-inductance and coupling factor of the coils, hence the mutual inductance. The characteristics of the designed coil are shown in Table 1.

### 2.1. Metal Barrier Effect

**Figure 2:**
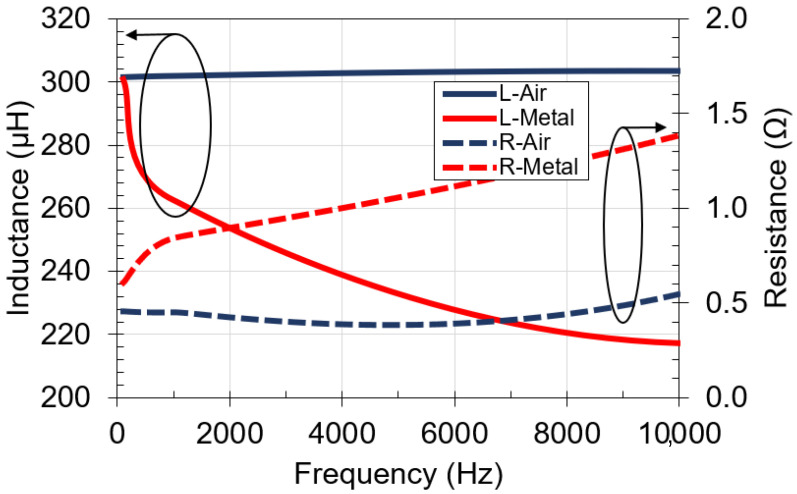
Measured inductance and resistance versus frequency for coil on: air and 1 mm-thick aluminum plate.

Figure 2 shows measured results of inductance, *L*, and resistance, *R_s_*, of the designed coil in two different scenarios: when the coil is located on top of a nonmetallic surface (L,R-Air), and when it is located on top of a 1 mm-thick aluminum plate (L,R-Metal). Measurements were made in the frequency range from 500 Hz to 10 kHz using a passive component LCR meter (LCR200 from Extech). For the case when the coil is located on top of a nonmetallic surface, inductance and resistance remain fairly constant with respect to frequency, L=300 μH and $R_{s}=0.4\;\Omega$. On the contrary, when the coil is located on top of the metal plate, inductance decreases (L=216 μH) with frequency due to the induced opposite current on the plate that cancels the current from the coil [20]. Likewise, the resistance increases ($R_{s}=1.4\;\Omega$) because the induced current on the plate contributes to the total resistance of the coil.

**Figure 3:**
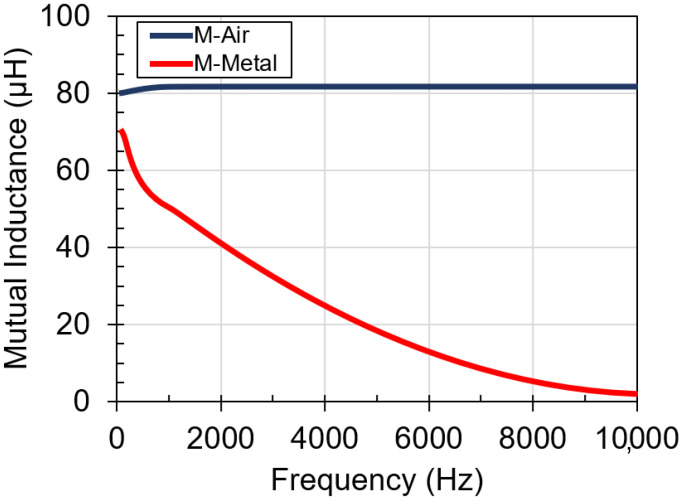
Mutual inductance versus frequency for coil on: air and 1 mm-thick aluminum plate.

Figure 3 shows the effect of the metal plate on mutual inductance. There is a significant decrease in mutual inductance with respect to frequency, from 80 μH to 2.5 μH. The mutual inductance is equal to the product of self-inductance and coupling factor k. As the self-inductance drops with respect to frequency, the mutual-inductance also decreases as shown in Figure 3. Additionally, the skin effect from the metal plate will lower the coupling factor k [21]. Thus, the mutual inductance will decrease further, limiting the upper operating frequency of the wireless power transfer system.

### 2.2. Load Effect

**Figure 4:**
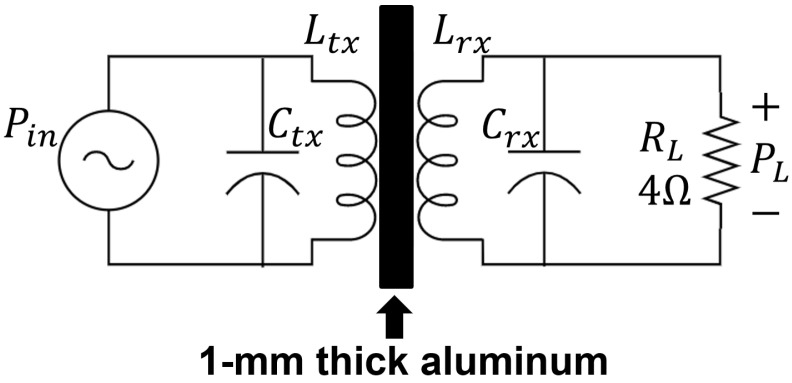
Circuit used to measure harvested power for the designed coil.

**Figure 5:**
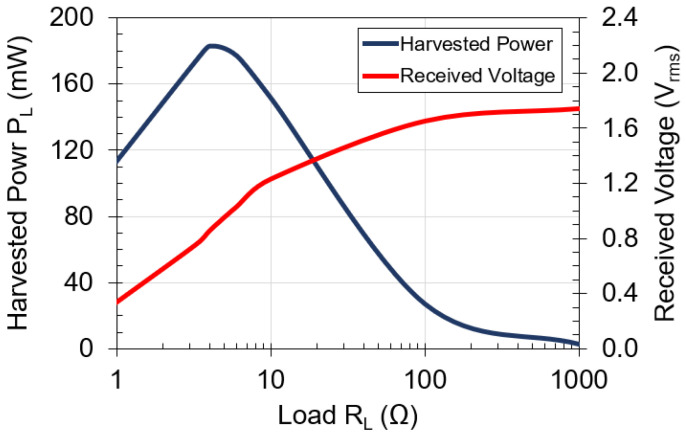
Harvested power and received voltage versus load for designed coil with 1 mm-thick aluminum plate in between. Operating frequency is 2 kHz. Input power is 8.5 W.

The miniature coil was designed to have an input resistance of a few ohms that will allow efficient rectification and matching while providing high harvested power [22]. Figure 4 shows the circuit diagram used for measuring harvested power. Parallel capacitors, $C_{tx}=C_{rx}=24\;\mu F$, are used to resonate the system at 2 kHz to maximize power transfer [23]. Load $R_{L}$ was varied in the range 1 Ω–1 kΩ to find an optimal value. Figure 5 shows measured results of harvested power and received voltage for different loads. The optimal load is 4 Ω and harvests 180 mW with 8.5 W of input power. As load $R_{L}$ rises, the received voltage increases while the harvested power decreases. At a 4 Ω load, the harvested power peaks at 180 mW, while the received voltage is 850 mV_rms_, which should be high enough to achieve rectifier efficiencies between 45–62% [24]. The 4 Ω load provides an optimal impedance match at the output of the receiving coil.

### 2.3. Coil Performance

**Figure 6:**
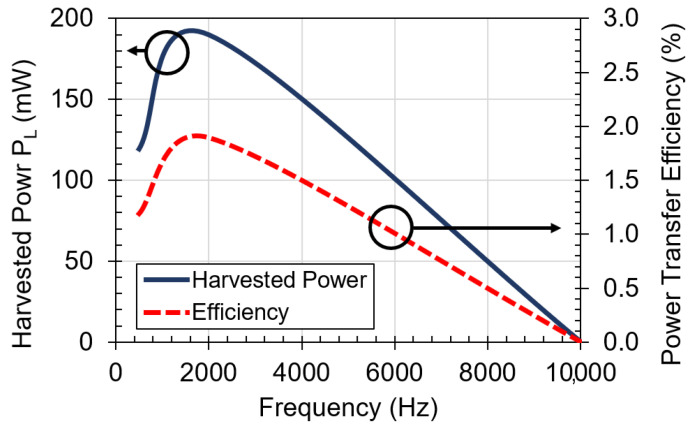
Measured harvested power versus frequency for designed coil with 1 mm-thick aluminum plate in between. Input power is 10 W.

Figure 4 shows the circuit diagram used to measure the performance of the designed coil. For each operating frequency, from 1–10 kHz, the values of the parallel capacitors were changed to ensure the resonance condition and harvested power was measured. Figure 6 shows measured results for harvested power and power transfer efficiency versus frequency. The maximum harvested power using this configuration is 190 mW at 2 kHz with an input power of 10 W. At 2 kHz, the parallel capacitances to create resonance are $C_{tx}=C_{rx}=24\;\mu F$.

**Figure 7:**
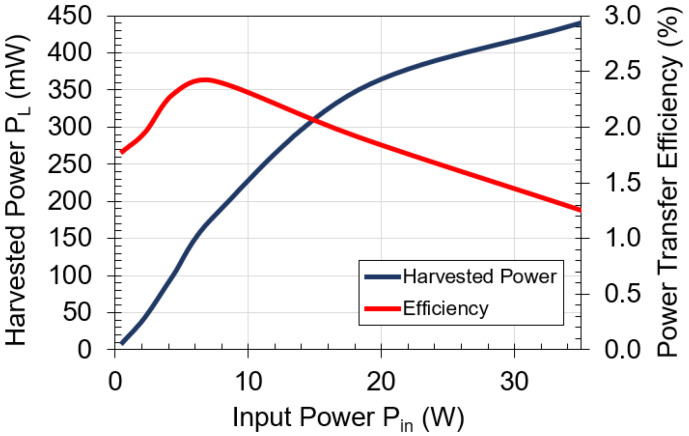
Measured harvested power and power transfer efficiency versus input power for the designed coil with 1 mm-thick aluminum plate in between. Operating frequency is 2 kHz.

Figure 7 shows the measured harvested power and power transfer efficiency versus input power at the optimal frequency of 2 kHz. The coil is designed to handle up to 35 W input power at the transmitting side so that the receiver coil can harvest up to 440 mW. The power transfer efficiency varies from 2.5% to a minimum of 1.2% at the highest input power of 35 W. The input power limit of 35 W is related to the maximum current and temperature ratings of 24 AWG wires from the manufacture datasheet [25,26]. At 35 W input power, the measured temperature of the coil temperature using a thermocouple is 110 °C, which is below the rated operating temperature of 200 °C for 24 AWG wires specified by the manufacturer. The coil is separated 0.5 mm away from the metal plate at each side of the barrier.

Table 2 shows a survey of similar works on energy transfer through aluminum and stainless steel. As seen from Table 2, coils used for through metal energy transfer are large and their volumes range from 21,205 mm^3^ to 3,619,114 mm^3^. The reported frequency of operation for through metal energy transfer is below 500 Hz. Our frequency is much higher, at 2 kHz, and our coil size of 15 mm × 13 mm × 6 mm and volume of 1170 mm^3^ are the lowest. Our energy transfer per volume is 0.376 mW/mm^3^ which is very high compared to other works.

## 3. Through Metal Scenarios

**Figure 8:**
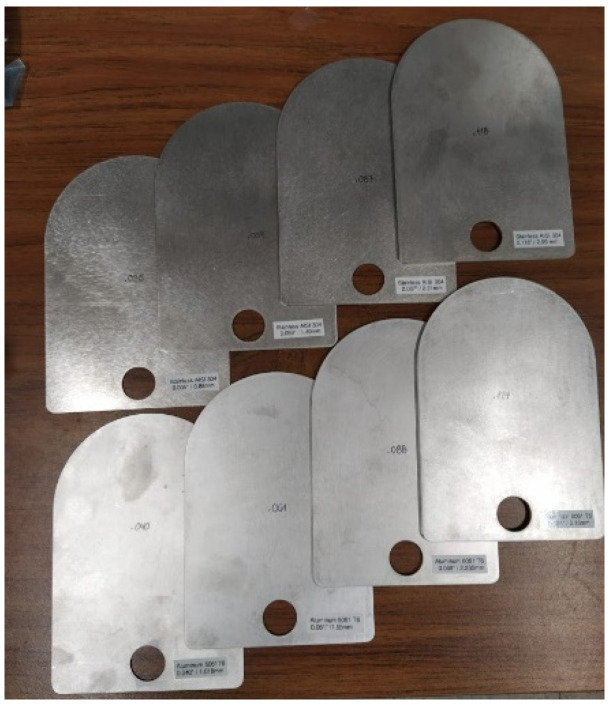
Plates of different materials used in the experiments.

This section includes the performance of the coil, in terms of harvested power and power transfer efficiency, for different types of materials, thicknesses and frequencies. Figure 8 shows a picture of the different plates used in the experiments. Two types of alloys are used: stainless steel (AISI 304) and aluminum (6061T6). Different thicknesses of each of the plates are summarized in Table 3. In these experiments, we place one coil on each side of a plate to form an inductive link as shown in Figure 4.

### 3.1. Stainless Steel

**Figure 9:**
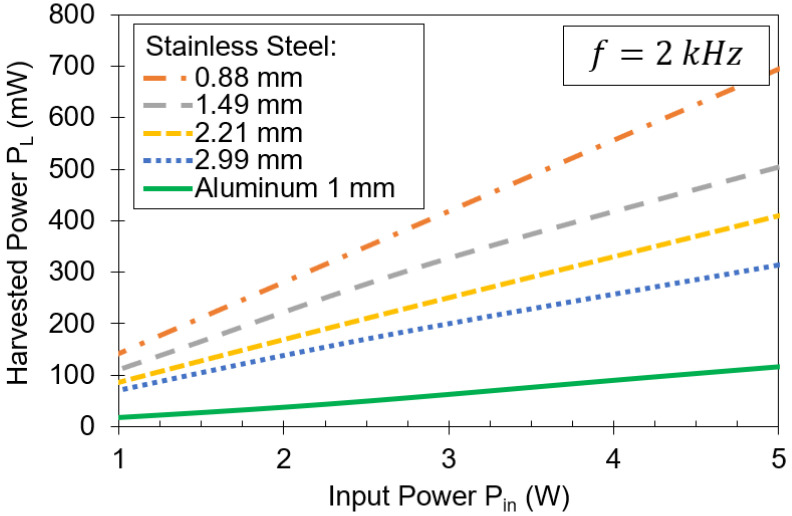
Harvested power versus input power using stainless steel plates of different thickness. Operating frequency is 2 kHz.

Figure 9 shows the measured results of harvested power versus input power for stainless steel plates of different thicknesses. Stainless steel is a material that is less conductive than aluminum. The conductivity of stainless steel and aluminum is $1.38\times 10^{6}\;S/m$ and $25\times 10^{6}\;S/m$, respectively [29]. Hence, the skin effect is reduced and power can be transferred more effectively through stainless steel [30]. For a 0.88 mm-thick stainless steel plate, the harvested power is almost 700 mW. If the plate thickness is increased to 1.49 mm, more than 400 mW can be harvested. When using a 2.99 mm-thick stainless steel plate, 300 mW can be harvested, which is nearly double the harvested power through a 1 mm-thick aluminum barrier.

**Figure 10:**
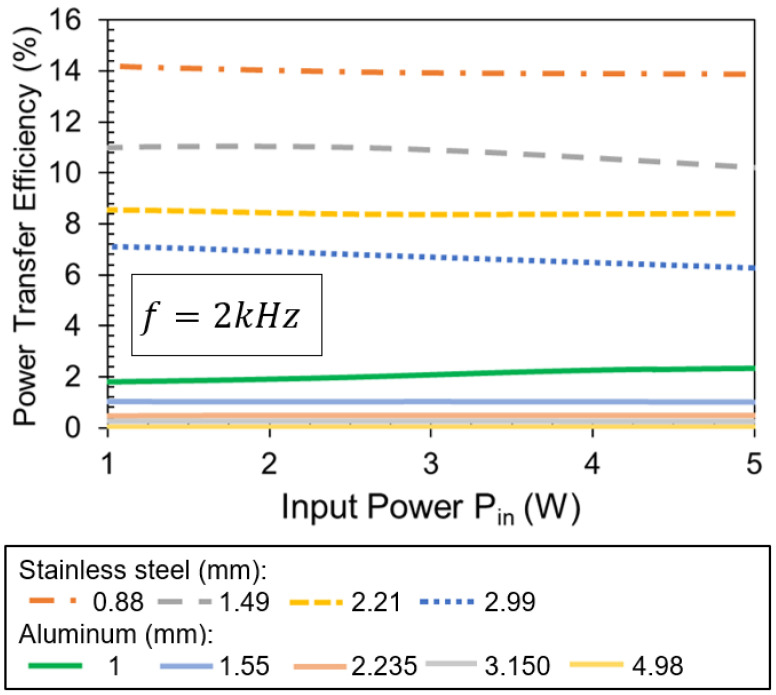
Power transfer efficiency versus input power using stainless steel and aluminum plates of different thickness. Operating frequency is 2 kHz.

Figure 10 shows the power transfer efficiency results for the same configuration. By using stainless steel, efficiency can be tripled, even if the thickness of the plate is 2.99 mm. Efficiency increases further with thinner plates. For stainless steel, power transfer efficiency can go from a minimum of 6% to a maximum of 14% for thicknesses from 2.99 to 0.88 mm, respectively. Figure 10 also includes the power transfer efficiency results for aluminum plates of different thicknesses. As the aluminum plate thickness increases from 1.5 to 4.98 mm, the efficiency decreases from results 1% to 0.04%, respectively. For the thickest aluminum plate, 4.98 mm, less than 0.04% efficiency is obtained.

The skin depth ($\delta =1/\sqrt{\pi f\mu \sigma }$) [31] of stainless steel and aluminum at 2 kHz is 9.57 mm and 2.25 mm, respectively. Therefore, the E-field $\left(E=E_{0}\;e^{-d/\delta }\right)$ attenuated into the metal faster for aluminum than stainless steel. In other words, at the same distance into metal, the electric field amplitude in stainless steel is much higher than that in aluminum. As a result, harvested power through stainless steel is higher than that through aluminum for the same metal thickness.

**Figure 11:**
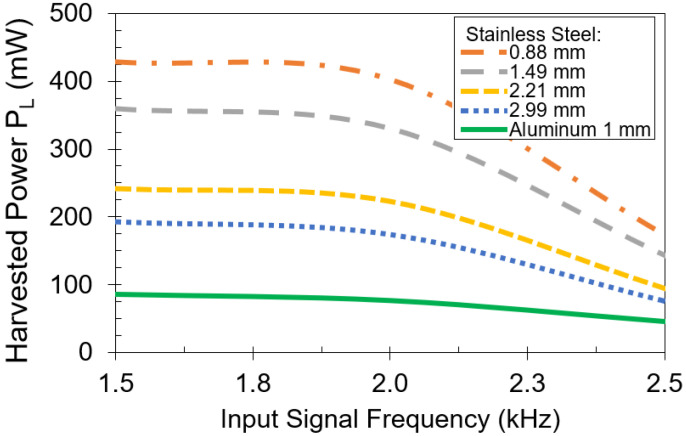
Harvested power versus frequency using stainless steel plates of different thicknesses. Input power is 3 W.

Figure 11 describes the measured harvested power with respect to frequency at an input power of 3 W for stainless steel. In this experiment, the frequency of the input signal is swept from 1.5 kHz to 2.5 kHz while load $R_{L}=4\;\Omega$ and parallel capacitances $C_{tx}$ and $C_{rx}$ are fixed at 24 μF. The results indicate that harvested power starts to decrease above 2 kHz due to the skin effect of the metal plate. In addition, above 2 kHz, the circuit in Figure 4 is no longer operating at resonance. To achieve maximum harvested power, the resonance principle should be maintained [32].

**Figure 12:**
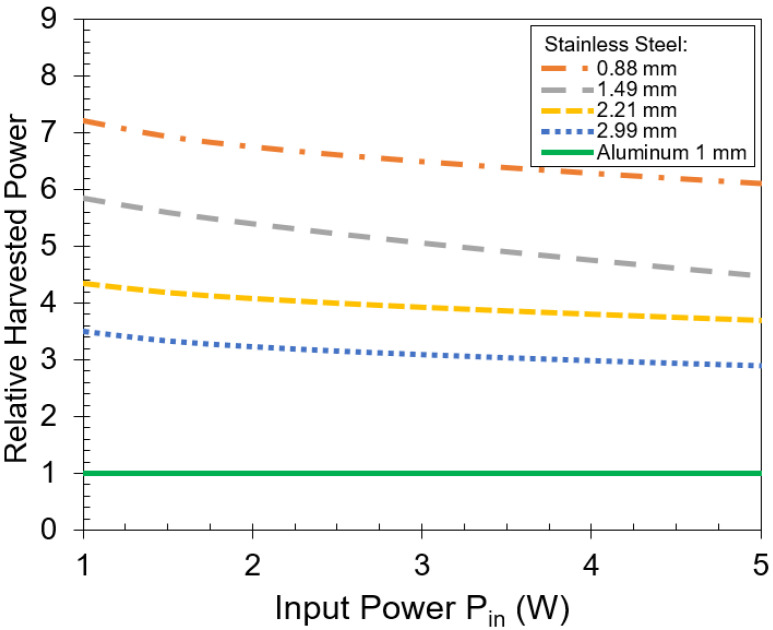
Relative harvested power through stainless steel plates to through a 1 mm-thick aluminum plate versus input power. The frequency is 2 kHz.

Figure 12 compares the harvested power through different stainless steel plates relative to the 1 mm-thick aluminum. The harvested power through a 2.99 mm-thick stainless steel plate is three times more than through a 1 mm-thick aluminum. As the thickness of the stainless steel plate decreases to 0.88 mm, the harvested power increases 7 times more than that through 1 mm-thick aluminum.

### 3.2. Aluminum

**Figure 13:**
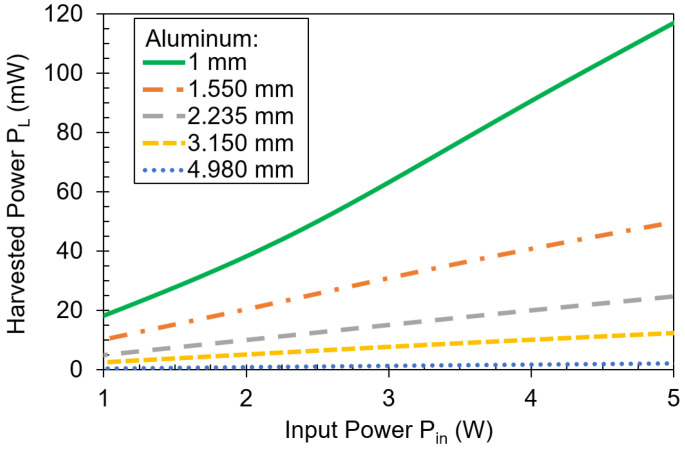
Harvested power versus input power using aluminum plates of different thickness. The frequency is 2 kHz.

Figure 13 shows the measured results of harvested power through aluminum plates of different thicknesses. Aluminum is more conductive than stainless steel [33], so the harvested power is considerably decreased as the thickness of the plates increases from 1 mm. For a 1.55 mm-thick plate, the harvested power is only 74 mW, less than half of the harvested power using a 1 mm-thick aluminum plate. If the plate thickness is increased to 2.235 mm, 36 mW are available. When the thickness is 3.15-mm, almost three times the thickness of the base line plate of 1-mm-thick aluminum, only 17 mW are available. Further increasing the barrier to 4.98 mm makes the harvested power limited to less than 3 mW. In conclusion, stainless steel is much better than aluminum for power transfer and signal communication through it.

## 4. Through Metal Communication System

**Figure 14:**
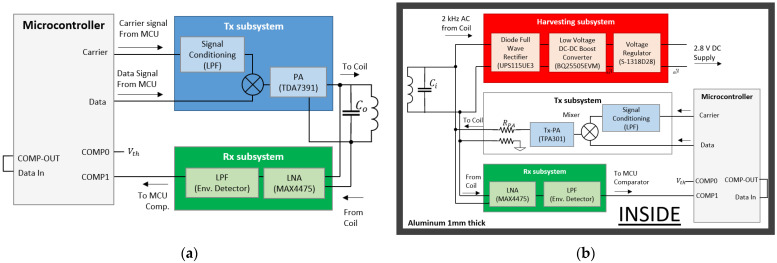
Fully integrated communication system: (**a**) outside, and (**b**) inside subsystem.

Figure 14 shows the fully integrated communication system for a through metal application. Here, two distinct subsystems can be identified: outside, and inside subsystems. The outside subsystem will wirelessly transfer power to the inside subsystem through the metal plate to enable its operation. Bidirectional communication is possible using only the harvested power from outside. There are no batteries used inside the metal barrier. Only one pair of miniature coils is used for both energy transfer and wireless communications.

### 4.1. Outside Subsystem

The outside subsystem is shown in Figure 14a and is made of a Tx subsystem, Rx subsystem and microcontroller, the ATtiny104 Xplained Nano from Microchip Technology. The Tx subsystem includes a transistor-based mixer, part number BC547 from Fairchild, and a power amplifier, part number TDA7391 from STMicroelectronics, to transmit the power and data signals through the metal plate. The Rx subsystem uses a low noise amplifier to recover the signal and an envelope detector together with a comparator for demodulation. The low noise amplifier, part number MAX4475 is from Maxim Integrated configured for gain >400. The envelope detector was based on a passive design, and the comparator uses the built-in module of the microcontroller. When the Tx subsystem delivers its full power in the WPT mode, the low noise amplifier is turned off to avoid overloading. In the Rx mode, the low noise amplifier is turned on while the power amplifier is off.

### 4.2. Inside Subsystem

The inside subsystem is made of a harvesting subsystem, Tx subsystem, Rx subsystem and microcontroller ATtiny104 Xplained Nano from Microchip Technology. Communication is based on amplitude shift keying (ASK) modulation due to its simplicity for wireless data transfer through metal [34]. Figure 14b shows the Tx Subsystem which consists of a low power mixer using a NPN transistor, part number BC547 from Fairchild, and the power amplifier, part number TPA301 from Texas Instruments. The Rx subsystem uses a low noise amplifier, part number MAX4475 from Maxim Integrated, a passive envelope detector, and the built-in comparator module of the microcontroller for demodulation.

Figure 14b shows the harvesting subsystem used to rectify and regulate the harvested AC power from outside to supply DC power for the operation of the inside subsystem. The harvesting subsystem consists of a Schottky diode full wave rectifier, UPS115UE3 from Microchip Technology, to convert the AC harvested signal to DC. Using this full wave rectifier, the total forward voltage drop is $V_{F}<0.2\;V$. The voltage at the output of the rectifier is only 1 V_rms_ and needs to be upscaled to at least 4 V_rms_ to charge the storage capacitor and supply power to the inside subsystem. To boost the voltage, a low-voltage boost converter, BQ25505 from Texas Instruments, was used. The boost converter has efficiency of >80%. Finally, a voltage regulator, S-1318D28 from ABLIC, was used to supply a constant 2.8 V supply for the inside communication subsystem for operation. The voltage regulator has a current consumption $I_{ss}<100\;\mathrm{nA}$.

**Figure 15:**
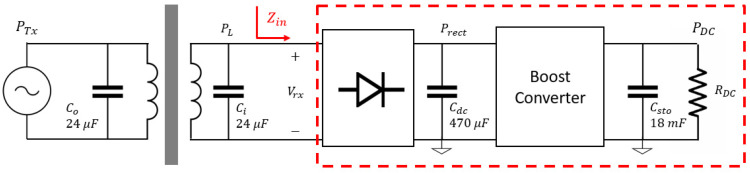
Complete schematic of harvesting subsystem.

Figure 15 shows the input impedance $Z_{in}$ seen by the inside coil. Impedance $Z_{in}$ has an important effect in received voltage, rectifier efficiency and harvested power [35]. To measure received voltage, rectifier efficiency and harvested power, the outside transmitted power, $P_{Tx}$, was fixed to 35 W at 2 kHz. The coil setup was the same for the rest of this paper, and the barrier is a 1 mm-thick aluminum plate. Additionally, the load $R_{DC}$ on the inside subsystem was modified in the range of 10–1200 Ω to change equivalent input impedance $Z_{in}$. The impedance $Z_{in}$ was calculated by measuring the voltage and current at the input of the full wave rectifier.

**Figure 16:**
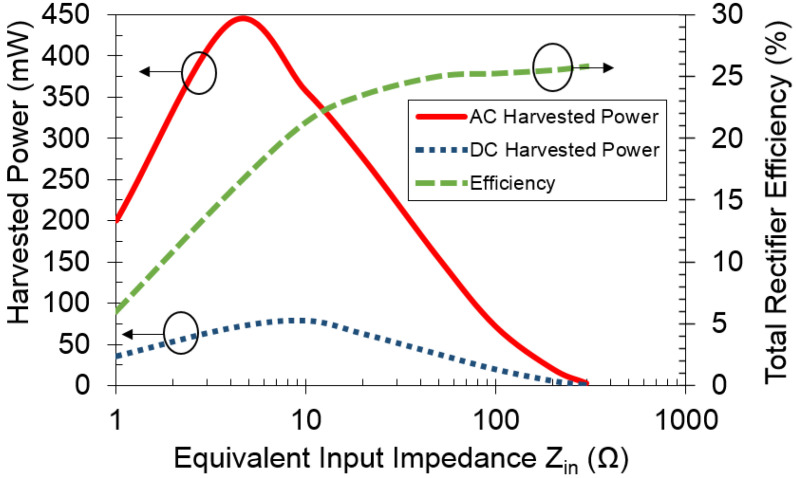
Measured harvested AC and DC power and total efficiency versus equivalent input impedance $Z_{in}$.

Figure 16 shows the harvested AC and DC power and total rectifier efficiency versus equivalent input impedance $Z_{in}$. When the equivalent input impedance $Z_{in}$ is between 1–10 Ω, the harvested AC power ranges from 200–356 mW with a peak of 440 mW at 4 Ω. In the same impedance range, the total rectifier efficiency is between 6% to 21% due to the low received voltages, typically $V_{rx}=0.5\;V_{rms}$. As a result, the harvested DC power goes from 6 mW to a maximum of 75 mW at 10 Ω. Input impedance $Z_{in}=10\;\Omega$ is the optimal value that provides a balance in rectifier efficiency and AC harvested power. When input impedance increases further, $Z_{in}\geq 10\;\Omega$, the total rectifier efficiency increases to almost 25% while the AC harvested power degrades rapidly. Hence, when $Z_{in}\geq 10\;\Omega$, the DC harvested power is reduced to 1 mW.

### 4.3. Prototype

**Figure 17:**
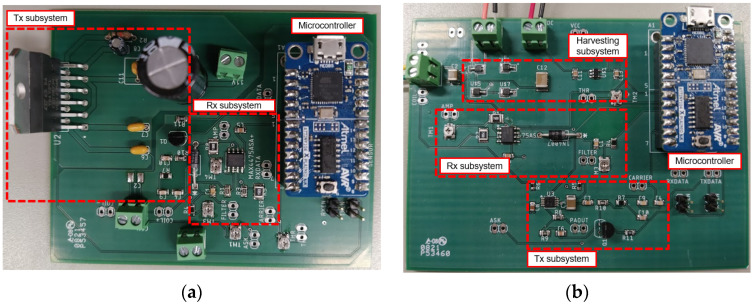
Built prototypes for wireless power transfer through metal communication system: (**a**) outside, and (**b**) inside subsystems.

The built prototypes for the outside and inside subsystems are shown in Figure 17. A communication protocol for this specific application was designed to enable automatic power transfer to the inside subsystem and bidirectionally data communications using one pair of coils. Four different operation modes were defined: Wireless Power Transfer (WPT), Tx, Rx and Sleep. The operation of the system is as follows:

1. Outside subsystem enters WPT mode, and transmits a high-power signal to charge the inside subsystem through the 1 mm-thick aluminum plate.
2. Inside subsystem harvests energy. If enough power is available, it enters Rx mode and waits for an incoming data stream from the outside subsystem, otherwise toggles between Sleep and Rx modes.
3. Outside subsystem finishes its WPT mode and power transfer stops. Then, Tx mode begins and the outside subsystem transmits a data frame at a data rate of 100 bps.
4. Inside subsystem is in Rx Mode and demodulates the incoming data. If data is received correctly, the inside subsystem enters Tx mode.
5. Inside subsystem transmits data frame and goes back to Rx mode.
6. Outside subsystem is in Rx mode. The data is received and demodulated.
7. System is ready to start the cycle again.

Using this configuration, the outside subsystem transmits a power signal with a $P_{tx}=35\;W$ during 7 s to fully charge the inside subsystem. Then, power transfer stops and data are transmitted. At the inside subsystem, the receiver demodulates the incoming data and operates during 1 s with a power consumption of 19 mW. Then, it transmits data to the outside subsystem during 1 s with a power consumption of 39 mW. The inside subsystem consumes 240 µW in Sleep mode, 19 mW in Rx Mode, and 39 mW in Tx Mode. Using only the harvested power from outside, the inside subsystem can transmit and receive data for more than 2 s.

### 4.4. Experimental Validation

**Figure 18:**
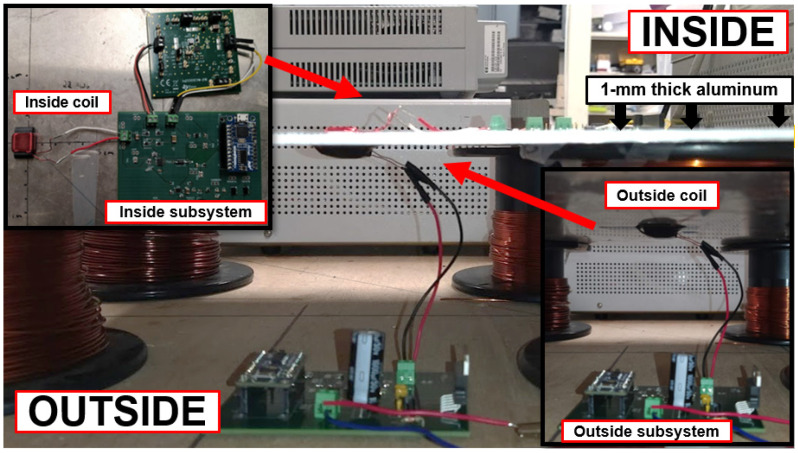
Test setup for through metal communication system. Below the metal plate: the coil connected to the outside subsystem. Above the metal plate: the coil and the inside subsystem. Each coil is located at the center of the 1 mm-thick aluminum plate and separated 0.5 mm away from the metal plate.

To validate our system, bidirectional communication was tested using the designed prototypes and a pair of coils. Each coil was located and aligned on each side of a 500 mm × 500 mm × 1 mm aluminum plate. The test setup is shown in Figure 18.

Using the previously described protocol, communication began at the outside subsystem with a push of a button of the microcontroller. Then, the wireless power transfer signal, $P_{tx}=35\;W$, and data signal, 100 bps, were transmitted through the metal plate. The inside subsystem automatically harvested the power, processed the incoming data and transmitted a response. The outside subsystem processed the data and a LED lights up indicating the successful data reception. Using this procedure, successful communication outside–inside and inside–outside was confirmed. The system operated using only the harvested power through the metal plate.

**Figure 19:**
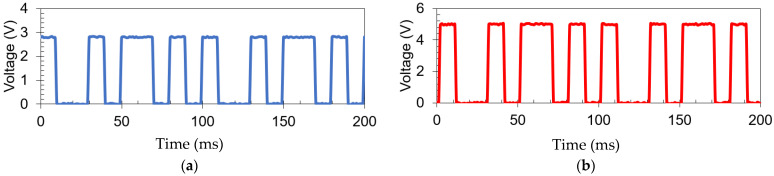
Transmit (**a**) and receive (**b**) data signal at 100 bps using a 2 kHz carrier.

For further validation, a sample of inside to outside data transfer is presented in Figure 19. The transmit data corresponds to the digital output of the microcontroller of the inside subsystem, and the receive data is the demodulated data at the output of the outside subsystem. Communication is successful at a data rate of 100 bps using a 2 kHz carrier. A bit error rate analysis (BER) was calculated for the data transmission through metal using 360,000 bits during a time period of 60 min of data transmission. The results showed that the BER < $1\times 10^{-6}$ for the wireless power transfer data link through metal.

## 5. Conclusions

This paper presents the first demonstration of power and data transfer through metal using a miniature coil. The system can operate through a 1 mm-thick aluminum plate using a coil of very small size, only 15 mm × 13 mm × 6 mm. The maximum coil-to-coil power transfer efficiency is 2.4%, and the maximum harvested power is 440 mW operating at 2 kHz. Additionally, the designed coil can harvest power in a variety of materials of different thicknesses. Harvested power is more than 10 mW through a 4.98 mm-thick aluminum plate, and more than 4.5 W through a 0.88 mm-thick stainless steel plate.

## Figures and Tables

**Table 1:** Electrical characteristics of the designed coil.

Frequency (kHz)	Inductance L (µH)	Quality Factor Q
1	301.9	4.195
10	303.5	34.75

**Table 2:** Survey of similar works on energy and communications through aluminum and stainless steel.

Reference	Coil Size (mm)	Metal Barrier Thickness	Harvested Power (mW)	Coil Power Transfer Efficiency (%)	Frequency (Hz)	Volume (mm^3^)
[9]	30 × Ø30	Tin 0.5 mm	0.03	-	50	21,205
[4]	234 × 67 × 30	Stainless steel 1.6 mm	3000	43	50	470,340
[27]	80 × Ø240	Aluminum 4 mm	18,181	23	100	3,619,114
[28]	8 × Ø115	Stainless Steel 20 mm	7000	5.5	500	83,095
[12]	50 × Ø98	Aluminum 3.1 mm	5000	9	250	377,148
This work	15 × 13 × 6	Aluminum 1 mm	440	2.5	2000	1170

**Table 3:** Plates used for the experiments.

Stainless Steel AISI 304 Thickness (mm)	Aluminum 6061T6 Thickness (mm)
0.88	1.55
1.49	2.235
2.21	3.15
2.99	4.98

## Data Availability

The data presented in this study is available from the corresponding author upon reasonable request.
